# Milk and meat safety in Nepal: addressing challenges and exploring solutions

**DOI:** 10.1016/j.soh.2025.100116

**Published:** 2025-06-28

**Authors:** Deepak Subedi, Sameer Thakur, Anil Gautam, Madhav Poudel, Sumit Jyoti, Abhinandan Devkota, Milan Kandel, Ananda Tiwari

**Affiliations:** a Department of Poultry Science, University of Georgia, Athens, GA 30602, USA; b Animal Service Department, Dhangadhi Sub-Metropolitan City Office, Dhangadhi 10900, Nepal; c Paklihawa Campus, Institute of Agriculture and Animal Science, Tribhuvan University, Rupandehi 32900, Nepal; d Faculty of Animal Science, Veterinary Science and Fisheries, Agriculture and Forestry University (AFU), Rampur 44209, Chitwan, Nepal; e Department of Health Management, Atlantic Veterinary College, University of Prince Edward Island, Charlottetown, PEI C1A 4P3, Canada; f College of Agriculture, Engineering, Medical and Veterinary Science, Nepal Polytechnic Institute, Purbanchal University (PU), Bharatpur 44200, Chitwan, Nepal; g School of Life and Environmental Sciences, Faculty of Science, The University of Sydney, Camden, NSW 2570, Australia; h Poultry Research Foundation, The University of Sydney, Camden NSW 2570, Australia; i Department of Food Hygiene and Environmental Health, Faculty of Veterinary Medicine, University of Helsinki, Helsinki 00014, Finland

**Keywords:** Bacteria, Developing country, *Escherichia coli*, Food safety, Nepal, Public health, *Salmonella*

## Abstract

The transmission of zoonotic diseases through animal-derived food products poses a significant global public health challenge, with contaminated milk and meat serving as major transmission pathways. In Nepal, the growing consumption of these products has amplified the risk of foodborne illnesses, largely due to widespread bacterial contamination. This review systematically explores the prevalence, distribution, and public health significance of key bacterial pathogens, including *Salmonella, Escherichia coli*, *Shigella*, *Staphylococcus aureus*, *Brucella*, *Bacillus cereus*, *Mycobacterium tuberculosis*, and *Campylobacter* in Nepalese milk and meat products. The analysis identifies major contributing factors: inadequate hygiene and sanitation practices, weak regulatory frameworks, insufficient infrastructure, improper antibiotic usage, and limited public awareness. The high levels of bacterial contamination, coupled with the emergence of antibiotic-resistant strains, underscore the urgency for strategic interventions. Recommended measures include strict enforcement of hygiene and sanitation standards, strengthening regulatory policies, enhancing infrastructure, comprehensive public education campaigns, and prudent antibiotic stewardship. Implementation of these strategies is imperative to improve food safety, protect public health, and mitigate the risks posed by bacterial zoonotic diseases in Nepal.

## 1 Introduction

Zoonotic diseases, which are naturally transmitted between vertebrate animals and humans, represent a significant public health concern worldwide. Alarmingly, more than 60 % of all infectious diseases and 75 % of emerging infectious diseases are estimated to be of zoonotic origin [1]. These diseases are typically transmitted through direct contact with infected animals [2] or humans [3], consumption of contaminated food or water [4], and contact with contaminated environments, fomites, or vectors [5]. Foodborne zoonoses are acquired through the consumption of animal-derived products like unpasteurized milk, raw or undercooked meat, and contaminated eggs [6,7]. These diseases pose a significant risk to public health and disrupt the production and trade of animal products for food and other purposes [8]. According to the World Health Organization (WHO), unsafe food causes 600 million illnesses and 420,000 deaths annually, with children under five accounting for 30 % of these fatalities [9]. Around 10 % of the global population falls ill each year due to the consumption of contaminated food, with milk and meat accounting for the largest share of these cases [10].

In Nepal, the rising consumption of milk and meat, currently averaging 79 L and 18 kg per capita per year, respectively, reflects the rapid growth of the billion-rupee livestock industry [11]. However, this increase in demand has not been matched by commensurate improvements in hygiene practices, regulatory oversight, or food safety infrastructure [12]. The lack of modern slaughterhouses and cold chain infrastructure, poor milk and meat handling practices, environmental contamination, and the widespread use of antibiotics have all contributed to significant bacterial contamination in the milk and meat supply [13,14]. Numerous studies from Nepal have reported alarming levels of bacterial contamination in milk and meat products, including the presence of foodborne pathogens like *Salmonella*, *Escherichia coli*, *Shigella*, *Staphylococcus aureus*, *Campylobacter*, *Brucella*, *Bacillus cereus*, and *Mycobacterium tuberculosis* [[13], [14], [15], [16], [17]]. These bacteria are responsible for significant public health burdens, especially among vulnerable populations. The situation is further compounded by the misuse of antibiotics in food animals, which has contributed to the emergence and spread of antimicrobial-resistant pathogens, posing additional threats to both human and animal health [18]. Despite the presence of food safety standards, including the *Food Act (1967)*, the *Animal Slaughterhouse and Meat Inspection Act (1999)*, and the *Food Safety Policy (2019)*, enforcement remains weak due to limited infrastructure, technical capacity, and public awareness [14,17]. Traditional practices, including the consumption of raw milk and undercooked meat, further increase the population's exposure to foodborne pathogens.

Given these multifaceted challenges and public health implications, a comprehensive understanding of the extent and nature of bacterial contamination in milk and meat is essential. Although numerous studies have reported the presence of bacterial pathogens in milk and meat, the data remain scattered, often lacking integration across microbial, regulatory, and socio-cultural domains. This review critically examines the prevalence, types, and public health significance of bacterial pathogens in milk and meat supply chains in Nepal. It also assesses the risk factors and challenges associated with food safety and outlines targeted strategies for improving hygiene practices, regulatory compliance, antibiotic stewardship, and public education. The outcomes are intended to inform science-driven policy formulation, enhance national food safety strategies, and support the development of a resilient, health-conscious livestock production system in Nepal.

## 2 Milk and meat production in Nepal

**Fig. 1:**
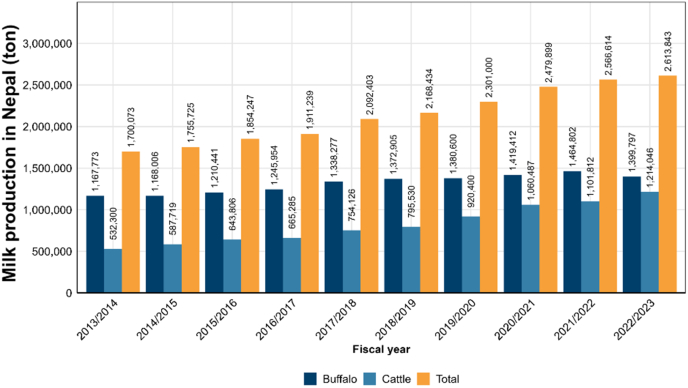
Milk production in Nepal over the past decade from the fiscal year 2013/2014 to 2022/2023.

Over the decade from 2013/2014 to 2022/2023, national milk production experienced a notable upward trajectory, with total output rising by 53.8 %, from 1.70 million t to 2.61 million t (Fig. 1) [19]. This represents an average annual growth rate of 5.4 %. Cattle milk production accounted for the most significant increase, more than doubling from 532,300 t to 1,214,046 t—an overall rise of 128.1 % and an average annual growth rate of 12.8 %. In contrast, buffalo milk production demonstrated a relatively modest growth of 19.9 %, increasing from 1,167,773 t to 1,399,797 t over the same period, with an average annual increase of 2 %. While buffalo milk still represents a larger share of national milk output, the proportion of cattle milk has risen considerably. This shift highlights evolving trends in dairy production systems, driven primarily by breed improvement, changes in feed and management practices, market preferences, and policy support favoring higher-yielding cattle breeds [20].

**Fig. 2:**
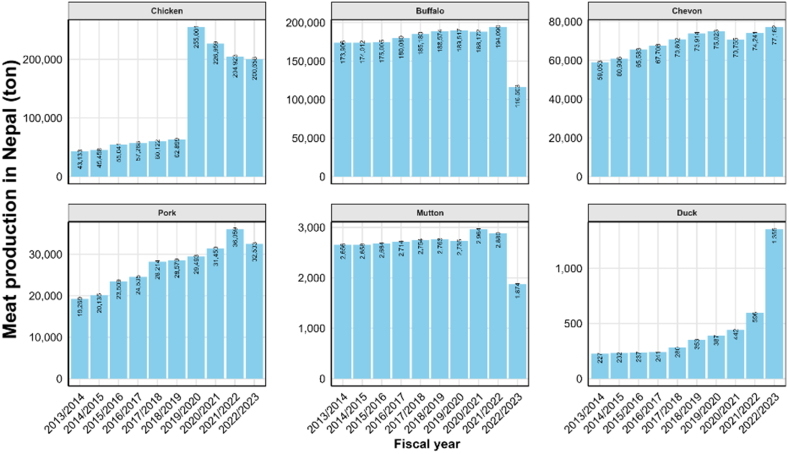
Meat production in Nepal over the past decade from the fiscal year 2013/2014 to 2022/2023.

Between 2013/14 and 2022/23, total meat production (buffalo, mutton, chevon, pork, chicken and duck) in Nepal increased by 44.20 %, from 298,244 t to 430,085 t, with an average annual growth rate of 4.40 % (Fig. 2) [19]. However, trends varied by meat types. Poultry showed the most substantial growth: chicken surged by 365.30 % (from 43,133 t to 200,658 t), with an average annual increase of 36.52 %, while duck meat surged by 496.50 % (from 227 t to 1355 t), growing 49.65 % annually. In contrast, red meats such as buffalo and mutton production declined. Buffalo meat dropped by 33.01 % (from 173,906 t to 116,503 t), and mutton by 29.40 % (from 2656 t to 1874 t), with negative annual growth rates of −3.30 % and −2.90 %, respectively. Goat meat (chevon) increased moderately by 30.67 % (from 59,053 t to 77,162 t), averaging 3.10 % growth, while pork rose by 68.80 % (from 19,269 t to 32,533 t), averaging 6.90 % annual growth. These shifts reflect changing consumer preferences and production practices, with poultry gaining dominance, likely due to its shorter production cycle, lower input costs, and broader market appeal, while the large ruminant meat production is declining, possibly due to higher production costs, land constraints, and changing dietary trends. With increasing milk and meat consumption, this paper explores the associated food safety risks and challenges in Nepal.

## 3 Challenges of milk and meat safety in Nepal

### 3.1 Bacterial contamination of milk

#### 3.1.1 Salmonella

Salmonellosis is one of the most prevalent foodborne bacterial diseases globally [21]. In many developed nations, *Salmonella* ranks as the second most leading cause of foodborne illnesses, causing symptoms like diarrhea, abdominal cramps, vomiting, and fever [22]. In Nepal and other developing countries, however, it remains a neglected zoonotic disease [23]. A study by Parajuli et al. [24] found *Salmonella* in 7.5 % of 60 raw milk samples collected from local dairies (45 samples) and cow farms (15 samples) across the Kathmandu Valley. Another study examining 14 milk brands from valley outlets reported a 3 % contamination rate in raw milk samples [25]. The 2017 *Salmonella* *agona* contamination of Lactalis baby formula, linked to infant deaths across Europe, also raised global food safety concerns, including in Nepal [26]. Contributing factors to *Salmonella* contamination in milk likely include poor hygiene, use of contaminated utensils, and adulteration with unsafe water [27].

#### 3.1.2 Escherichia coli

Shiga toxin-producing *E. coli* (STEC), also known as verotoxin-producing *E. coli* (VTEC), is characterized by the presence of *stx*1 and *stx*2 genes [28]. This pathogen poses serious global public health and economic risks, primarily transmitted through contaminated milk, meat, and dairy products [29]. Symptoms of STEC infections range from mild diarrhea to severe illnesses like hemorrhagic colitis (HC) and hemolytic uremic syndrome (HUS), which occurs in 5 %–15 % of cases [30].

Numerous studies have reported *E. coli* contamination in milk across Nepal. One study found 92 % of raw milk samples from 14 brands in the Kathmandu Valley were contaminated [25]. Another reported contamination in 27.3 % of samples—18.8 % in pasteurized, 40 % in unpasteurized, and 20 % in raw milk (Table 1) [[30], [34]]. In Kathmandu district, 45.7 % of 70 milk samples (52.5 % raw, 36.7 % pasteurized) tested positive [33], while Parajuli et al. [24] found *E. coli* in 32 % of samples from local dairies and 24 % from cow farms in Kathmandu Valley. In Dharan, *E. coli* was detected in 55 % of raw and 30 % of pasteurized milk samples from five dairy industries [34]. Shrestha et al. [35] found 33.3 % of raw and 6.7 % of pasteurized samples contaminated in Kathmandu, Bhaktapur, Lalitpur, and Kavrepalanchok. Bohara et al. [36] reported a 43.3 % overall prevalence in Kathmandu, with 60 % of raw and 26.6 % of pasteurized samples testing positive. Gautam et al. [37] found *E. coli* in 29.4 % of 102 raw buffalo milk samples in Rupandehi. A recent study by Shrestha et al. [38] found *E. coli* in 29.6 % of 267 raw cattle milk samples, with STEC identified in 11.6 %. Among these, 1.1 % were O157 STEC and 10.5 % non-O157 STEC. The *z3276* gene, specific to O157, was found in only three isolates, all of which carried *stx* genes. These findings highlight the widespread contamination of milk with *E. coli*, including pathogenic STEC strains, across Nepal, underscoring urgent food safety challenges and the pressing need for stronger regulatory and hygiene measures.

**Table 1:** Prevalence of *Escherichia coli* in milk samples from various regions of Nepal.

Study location	Year	Milk type	Sample size	Microbiological test	*Escherichia coli* prevalence (%)	Reference
Kathmandu Valley and Kavrepalanchok districts	2023	Pasteurized and raw milk	30 (15 each)	Total viable count (TVC) by pour plate method, *E. coli* isolation and identification by inoculation on MacConkey agar	6.67 % for pasteurized and 33.33 % for raw milk	[35]
Kathmandu district	2021	Pasteurized and raw milk	30 (15 each)	Total coliform count by violet red bile agar (VRBA), *E. coli* isolation and identification by inoculation on MacConkey agar	Overall 43.3 %, 26.6 % for pasteurized, and 60 % for raw milk	[36]
Rupandehi district	2021	Raw buffalo milk	102	*E. coli* isolation and identification by inoculation on eosin methylene blue (EMB) agar, isolated colonies of *E. coli* were confirmed by Gram staining and biochemical tests	29.4 %	[37]
Chitwan district	2020	Raw cattle and buffalo milk	972 quarters from 243 animals (193 cows and 50 buffalo)	*E. coli* isolation and identification by inoculation on EMB agar, isolated colonies of *E. coli* were confirmed by Gram staining and biochemical tests	5 % (49/972)	[32]
Kathmandu district	2020	Pasteurized and raw milk	70 (30 pasteurized and 40 raw milk)	Total coliform count by pour plate method, *E. coli* isolation by enrichment in buffered peptone water and culture on EMB agar	Overall 45.71 %, 36.7 % for pasteurized, and 52.5 % for raw milk	[33]
Dharan district	2019–2020	Pasteurized and raw milk	40 (20 pasteurized and 20 raw milk)	Total plate count (TPC) by plate count agar, total coliform count (TCC) by VRBA, and thermoduric bacterial count (TBC), *E. coli* isolation and identification by inoculation on MacConkey agar	30 % for pasteurized and 55 % for raw milk	[34]
Kathmandu Valley	2019	Raw milk	60 (40 local dairies and 15 cow farms)	TPC by plate count agar, TCC by VRBA	32 %	[24]
Chitwan district	2018–2019	Raw cattle milk	267	*E. coli* isolation and identification by inoculation on MacConkey agar for non-O157 and sorbitol MacConkey (SMAC) agar for O157, confirmation with biochemical tests	Overall 29.6 % and 11.6 % for Shiga toxin-producing *E. coli* (STEC) (1.1 % for O157 STEC and 10.5 % for non-O157 STEC)	[38]
Kathmandu Valley	2017	Pasteurized, unpasteurized, and raw milk	66 (16 pasteurized, 25 unpasteurized, and 25 raw milk)	TPC, total coliform count by pour plate method, biochemical tests	18.8 % for pasteurized, 40 % for unpasteurized, and 20 % for raw milk	[31]
Kathmandu Valley	2004	Raw milk (14 different milk brands)	140 (10 samples of each brand)	Methylene blue reduction time test (MBRT), direct microscopic count (DMC), TPC	92 %	[25]

#### 3.1.3 Shigella

*Shigella,* commonly found in water and feces, can contaminate milk, causing bacillary dysentery, especially in developing countries [39]. Although a significant global health threat, *Shigella* is often overlooked in animals such as cows, chickens, pigs, and monkeys [40]. In countries like Nepal, shigellosis remains a major foodborne illness, particularly affecting children and immunocompromised individuals [41]. A study by Arjyal et al. [25] found *Shigella* in 3 % of raw milk samples from 14 milk brands in the Kathmandu Valley. Another study detected *Shigella* species, including *S*. *boydii*, *S*. *flexneri*, *S*. *sonnei*, and *S*. *dysenteriae*—in ice cream samples from Kathmandu, indicating poor hygiene and careless manufacturing practices [42]. However, a more recent study by Parajuli et al. [24] found no *Shigella* contamination in 60 raw milk samples from local dairies and cow farms in the Kathmandu Valley.

#### 3.1.4 Staphylococcus aureus

Staphylococcal food poisoning is a major global foodborne illness, second only to salmonellosis. *S*. *aureus*, a key pathogen in fresh and ready-to-eat foods, contaminates products through poor storage, handling, unsanitary utensils, and milking conditions, producing harmful enterotoxins that reduce food quality and safety [[43], [44]]. Studies from Nepal, especially in the Kathmandu Valley, report notable *S. aureus* contamination in milk. Arjyal et al. [25] found 15 % contamination in raw milk from 14 milk brands. Acharya et al. [31] reported 16 % contamination in unpasteurized, 12.5 % in pasteurized milk, and 24% in raw milk samples. Rai et al. [33] found *S. aureus* in 67.5 % of raw and 10 % of pasteurized milk samples (42.9 % overall). Another study reported contamination in 40 % of farm milk, 30 % of dairy milk, and 10 % of packaged milk (26.7 % overall) [18]. Bohora et al. [36] found 50 % contamination, mostly in raw milk (86.7 %). In Pokhara, Joshi et al. [45] found *S. aureus* in 29.7 % and methicillin-resistant *Staphylococcus aureus* (MRSA) in 11.25 % of 400 milk samples from 10 farms. In Dharan, prevalence was 45 % in raw and 20 % in pasteurized milk [34]. Tiwari et al. [46] reported an exceptionally high prevalence (93.3 %) in California mastitis test-positive raw buffalo and cattle milk in Chitwan. The prevalence of *S*. *aureus and* MRSA in milk samples from different regions of Nepal is summarized in Table 2.

**Table 2:** Prevalence of *Staphylococcus aureus* and methicillin-resistant *Staphylococcus aureus* (MRSA) in milk samples from different regions of Nepal.

Study location	Year	Milk type	Sample size	Prevalence (%)	Reference
Kathmandu district	2023	Farm milk	30	40 %	[18]
		Dairy milk	30	30 %	
		Pasteurized packaged milk	30	10 %	
Kathmandu Valley	2023	Raw milk	15	86.7 %	[36]
		Pasteurized milk	15	13.3 %	
Chitwan district	2022	Raw buffalo and cattle milk	104	93.3 %	[46]
Kathmandu district	2020	Raw milk	40	67.5 %	[33]
		Pasteurized milk	30	10 %	
Dharan district	2020	Raw milk	20	45 %	[34]
		Pasteurized milk	20	20 %	
Kathmandu Valley	2017	Pasteurized milk	–	12.5 %	[31]
		Unpasteurized milk	–	16 %	
Pokhara Valley	2014	Raw milk	400	29.7 % for *S. aureus* and 11.3 % for MRSA	[45]
Kathmandu Valley	2004	Raw milk	14 brands	15 %	[25]

#### 3.1.5 Brucella

Brucellosis is a major public health and livestock concern in developing countries, affecting both humans and animals [47]. Human infections are primarily caused by *Brucella abortus*, *Brucella melitensis*, *Brucella suis*, and *Brucella canis*, with *B. melitensis* being the most virulent, followed by *B. suis* [48]. As few as 10–100 organisms can cause infection. Transmission occurs mainly through unpasteurized milk, dairy products, or undercooked meat [49]. Symptoms are often non-specific—fatigue, malaise, arthritis, and fever—and while rarely fatal, the illness can be long-lasting and debilitating [50]. In animals, brucellosis impairs reproduction, reduces milk yield, and affects offspring survival [47]. National surveys by Nepal's Department of Health Services indicate a 2 %–3 % seroprevalence in cattle [17]. Although no studies have confirmed *Brucella* in raw milk or meat in Nepal, the high seroprevalence in livestock and human cases indicate a probable association.

#### 3.1.6 Bacillus cereus

*B.cereus* has emerged as a significant foodborne pathogen, frequently found in meat, milk, and dairy products [51]. Commonly found in soil, *B. cereus* likely enters the food chain through soil or water contamination [52]. It often causes outbreaks linked to heat-treated foods, where spore germination occurs. Without competing microbes, *B. cereus* proliferates post-cooking, producing enterotoxins that lead to illness [53]. Understanding its toxin profiles has clarified its role in foodborne disease [54]. Arjyal et al. [25] found *Bacillus* in 18 % of milk samples from 14 milk brands in Kathmandu Valley. Another study of 240 ready-to-eat foods in Kathmandu reported *B. cereus* in 40 % of the samples, with 28 % contamination in dairy products. Among 80 dairy samples, bacterial growth was found in 50 % of butter (3/6) and 37 % of cheese (19/52), while cream, ghee, and yogurt showed no growth [55].

#### 3.1.7 *Mycobacterium tuberculosis* and Mycobacterium bovis

Tuberculosis (TB) in humans is caused by *M*. *tuberculosis*, results in millions of infections and hundreds of thousands of deaths worldwide [56]. *M*. *bovis*, in contrast, causes bovine TB in cattle, also known as zoonotic TB, which humans can contract through unpasteurized dairy products, handling sick animals, or occupational exposure. The connection between zoonotic TB in humans and cattle in Nepal remains unclear [16]. Bovine TB is globally widespread, causing substantial economic losses in animal production. It is a major infectious disease in cattle, other domestic animals, and some wildlife, posing a public health risk through the consumption of raw milk, close proximity of cattle to human dwellings, and in individuals with immunosuppressive conditions [57]. Due to its severe impact on animal and human health, rigorous control measures are essential to mitigate the risk of *M*. *bovis* infection [50].

In Mahendranagar, Kanchanpur, researchers tested 70 symptomatic individuals (40 males and 30 females) from 125 households, analyzing a total of 200 samples. They found 10 cases positive for tuberculosis, indicating a 9 % prevalence rate (Table 3). To explore the potential for animal-to-human transmission, livestock from these households were also examined. Among the 70 livestock tested, 8 were positive, indicating an 11 % prevalence rate [58]. Additionally, a retrospective matched case–control study conducted at the National Tuberculosis Center (NTC) in Bhaktapur, Nepal, further explored the link between cattle exposure and human TB. The study found that cattle exposure was a significant risk factor for human TB, with 9.76 % of cattle testing positive by tuberculin, 37.4 % by the rapid test, and 5.7 % by ELISA, showing strong agreement between the tuberculin and ELISA results [16]. Similarly, a study conducted by Jha et al. [59] on milk and fecal samples from 36 buffaloes and 32 cattle in Kathmandu, all of which tested positive for the single intradermal cervical tuberculin (SICT) test, identified 36 mycobacterial strains. Of these, 13 strains were classified as *M. bovis*, affecting 17 % of buffaloes and 16 % of cattle. *M. bovis* was detected in both the milk and feces of one buffalo and one cattle, in the milk of three buffaloes and three cattle, and in the feces of two buffaloes and one cattle. The remaining 23 strains were found to be atypical mycobacteria [59]. In a study conducted by Pandey et al. [60] in Chitwan, 100 bovines (22 cattle and 78 buffalo) from 60 farms with tuberculosis-positive patients were tested for bovine tuberculosis. The overall prevalence was 15 % with 13.6 % of cattle and 15.4 % of buffaloes testing positive. Furthermore, 24 % of the tuberculosis patients reported consuming raw milk, suggesting that milk could be a potential channel for *M. bovis* transmission to humans [60]. More recently, a surveillance study by Upadhyaya et al. [61] involving 400 blood samples from cattle and buffalo found that 74 animals (18.75 %) tested positive for *M. bovis*, with the majority being cattle (71 samples, 17.75 %) and only three cases in buffalo (<1 %). This high prevalence of *M. bovis* in dairy animals poses a significant public health threat, especially to those consuming milk. These findings underscore the urgent need for improved hygiene, regulatory enforcement, pasteurization, and public education to mitigate the risk of milk-borne diseases.

**Table 3:** Prevalence of tuberculosis (TB) in livestock from various regions of Nepal.

Location	Year	Sample size	Test used	TB prevalence	Reference
Eastern Nepal	2023	400 (cattle and buffalo)	Rapid bovine TB test kit	18.75 % (17.75 % in cattle and 0.75 % in buffalo)	[61]
Kathmandu, Bhaktapur, Lalitpur and Kavrepalanchok districts	2020	123 cattle	Tuberculin, rapid test, ELISA	9.76 % of the cattle were positive by tuberculin, 37.4 % by the rapid test, and 5.7 % by ELISA	[16]
Chitwan district	2013	100 bovines (22 cattle and 78 buffalo)	Intradermal tuberculin test	15 % (13.6 % in cattle and 15.4 % in buffaloes)	[60]
Kathmandu district	2007	36 buffaloes, 32 cattle	Single intradermal cervical tuberculin (SICT)	17 % in buffaloes and 16 % in cattle	[59]
Mahendranagar and Kanchanpur districts	2003	70 humans, 70 livestock	Direct sputum examination in humans and intradermal tuberculin test in livestock	9 % in humans and 11 % in livestock	[58]

### 3.2 Bacterial contamination of meat

#### 3.2.1 Salmonella

*Salmonella* is a widespread pathogen affecting both humans and animals. In humans, salmonellosis often presents as a self-limiting form of food poisoning (gastroenteritis), but it can occasionally develop into a severe systemic infection (enteric fever) that necessitates immediate antibiotic treatment [62]. The zoonotic potential of *Salmonella* and the growing antimicrobial resistance (AMR) in its strains are important topics in scientific discussions. In Nepal, contaminated meat is a primary source of *Salmonella* infections in humans [63]. Major routes of contamination include fecal contamination from the gut and the use of substandard water during meat preparation and for cleaning chopping materials.

Research conducted in Nepal consistently shows that *Salmonella* is more prevalent in chicken meat compared to other types of meat, such as pork, buffalo meat, and chevon. For example, a study analyzing 123 raw meat samples from local markets in Kathmandu reported an overall prevalence of *Salmonella* in 11.4 % of the samples, with the highest detection rates in chicken meat (14.5 %), followed by buffalo meat (13.5 %), and chevon (3.3 %) (Table 4) [64]. Similarly, another study in Dharan found that 34 % of the 50 meat samples collected from local markets were tested positive for *Salmonella*, with chicken meat showing the highest prevalence at 60 %, followed by chevon (33.3 %), buffalo meat (20 %), and pork (10 %) [12]. Additionally, in another study involving 320 samples of chicken meat, chevon, buffalo meat, and pork (80 samples each), 26 samples were tested positive for *Salmonella*, resulting in an overall prevalence of 8.13 %. The specific prevalence rates were 10 % in both buffalo and chicken meat, 7.5 % in chevon, and 5 % in pork [65]. Moreover, in a study conducted in Bhaktapur Metropolitan City, 140 raw meat samples—70 each of buffalo and chicken—were tested, revealing a *Salmonella* prevalence of 8.6 % (6 out of 70) in both meat types [66]. Unhygienic slaughtering methods and environments significantly contribute to *Salmonella* contamination.

**Table 4:** Prevalence of *Salmonella* in meat samples from various regions of Nepal.

Study location	Year	Sample type	Sample size	*Salmonella* prevalence %	Reference
Chitwan district	2020	Chicken meat (fresh and frozen)	100	10 %	[68]
Bhaktapur district	2019	Raw meat (buffalo, chicken)	140 (70 each)	8.6 % (8.6 % in buffalo meat and 8.6 % in chicken meat)	[66]
Dharan city	2019	Chicken meat		35 %	[72]
Kathmandu Valley	2019	Chicken meat	81	16 %	[73]
Dharan city	2018	Raw meat (various types)	50	34 % (60 % in chicken meat, 33.3 % in chevon, 20 % in buffalo meat, and 10 % in pork)	[12]
Chitwan district	2017	Chicken meat		26.2 %	[71]
Kanchanpur district	2017	Fresh chicken meat	45	38 %	[74]
Pokhara Valley	2016	Raw meat (chicken, chevon, buffalo, and pork)	320 (80 each)	8.13 % (10 % in chicken meat, 10 % in buffalo meat, 7.5 % in chevon, and 5 % in pork)	[65]
Chitwan district	2013	Chicken meat		46.2 %	[69]
Chitwan district	2013	Chicken meat		18.8 %	[70]
Kathmandu district	2012	Environmental swabs (chopping boards, knives, and tables)	492 swabs	40.2 % (36 % in chopping boards, 32.9 % in knives, and 25 % in tables)	[67]
Kathmandu district	2006	Raw meat (various types)	123	11.4 % (14.5 % in chicken meat, 13.5 % in buffalo meat, and 3.3 % in chevon)	[64]

A study analyzing 492 environmental swab samples from 82 retail meat shops in Kathmandu found that 40.2 % of the shops were *Salmonella*-positive. The contamination rates were particularly high on chopping boards (36.0 %), knives (32.9 %), and tables (25.0 %) [67]. Another study conducted in Bharatpur, Chitwan, analyzed 100 chicken meat samples (70 fresh and 30 frozen) from wholesale and retail shops, revealing a 10 % prevalence of *Salmonella*. The consistent isolation rate across both fresh and frozen samples indicates that freezing does not eliminate contamination. This finding underscores the need for stricter hygiene practices and enhanced regulatory oversight to ensure food safety [68]. The prevalence of *Salmonella* in chicken meat varies across different regions of Nepal, with reported rates of 46.2 % and 18.88 % in the Chitwan district [[69], [70]], 26.2 % in Bharatpur Metropolitan [71], 38 % in Kanchanpur [53], 35 % in Dharan [72], and 16 % in the Kathmandu Valley [73].

#### 3.2.2 E. coli

*E*. *coli* are generally harmless bacteria that reside in the intestines of humans and animals, playing a role in maintaining intestinal health [75]. However, consuming food or water contaminated with certain strains of *E. coli* can lead to mild to severe gastrointestinal issues [76]. Some pathogenic strains, such as STECs, can cause life-threatening conditions [77]. Different *E. coli* strains are associated with various types of food and water contamination. *E. coli* O157 is commonly transmitted to humans through the consumption of contaminated food, particularly undercooked ground meat and raw milk [76]. Members of the Enterobacteriaceae family, such as *E. coli,* account for up to 40 % of foodborne illnesses caused by bacteria [78].

In Nepal, *E. coli* contamination in meat continues to pose a serious food safety concern, as consistently emphasized in scientific discussions. Among the published data from various regions of Nepal, the highest prevalence of *E. coli* contamination (100 %) was reported by Gautam et al. [73], who found all 81 raw chicken meat samples from retail shops in the Kathmandu, Lalitpur, and Bhaktapur districts to be positive. In the Kathmandu Valley, several studies have reported varying levels of *E. coli* contamination in raw meat. One study found a 10 % (4/40) prevalence in buffalo meat samples from local markets [79], while a similar study reported a 22.5 % (9/40) prevalence in chicken meat [80]. Additionally, research conducted in Bhaktapur Metropolitan City on 140 raw meat samples, comprising 70 buffalo and 70 chicken meat samples, revealed 34.3 % (24/70) *E. coli* prevalence in buffalo meat and 47.14 % (33/70) prevalence in chicken meat [66].

Studies conducted in the eastern region of Nepal also indicate a concerning level of *E. coli* contamination in meat samples. Research from a butcher's shop in Dharan involving 24 samples (6 chicken meat, 6 buffalo meat, 6 pork, and 6 chevon) found a 41.66 % incidence of non-sorbitol fermenting *E. coli* O157, with prevalence rates of 50 % in both chicken and buffalo meat, and 33.33 % in pork and chevon (Table 5) [81]. Another study in Dharan reported 54 % of the 50 meat samples (15 chicken meat, 15 pork, 10 buffalo meat, and 10 chevon) from local markets were contaminated with *E. coli,* with contamination levels of 66.6 % in chicken meat, 60 % in pork, 40 % in buffalo meat, and 46.7 % in chevon [12]. Similarly, Bantawa et al. (2019) found that 53 % of 83 meat samples (33 chicken meat, 27 pork, 13 buffalo meat, and 10 chevon) collected from various meat shops in Dharan were positive for *E. coli* [72]. In Biratnagar, Morang, a study involving 80 meat samples from 40 outlets (40 chicken and 40 mutton) revealed an overall *E. coli* prevalence of 61.3 %, with 62.5 % in chicken and 60 % in mutton [82].

**Table 5:** Prevalence of *Escherichia coli* in meat samples from various regions of Nepal.

Study location	Year	Meat type	Sample size	*E coli* prevalence %	Reference
Chitwan district	2024	Chicken meat	105	58.1 %	[83]
Kathmandu Valley	2023	Chicken meat	40	22.5 %	[80]
Dharan city	2022	Mixed meat (chicken, buffalo, pork, and chevon)	24 (6 each)	41.66 % (*E. coli* O157)*,* 50 % in both chicken and buffalo, and 33.33 % in pork and chevon	[81]
Chitwan district	2022	Chopping board meat samples	–	33 %	[84]
Kathmandu Valley	2021	Buffalo meat	40	10 %	[79]
Chitwan district	2020	Chicken (fresh and frozen)	100	56 %	[68]
Kathmandu Valley	2019	Raw chicken meat	81	100 %	[73]
Bhaktapur district	2019	Raw meat (buffalo and chicken)	140 (70 each)	34.3 %	[66]
Dharan city	2019	Mixed meat (chicken, buffalo, pork, and chevon	83 (33 chicken, 27 pork, 13 buffalo, and 10 chevon)	53 %	[72]
Biratnagar district	2019	Chicken and mutton	80 (40 each)	61.3 % overall (62.5 % in chicken and 60 % in mutton)	[82]
Dharan city	2018	Mixed meat (chicken, buffalo, pork, and chevon	50 (15 chicken, 15 pork, 10 buffalo, and 10 chevon)	54 % overall (66.6 % in chicken, 60 % in pork, 40 % in buffalo, and 46.7 % in chevon)	[12]
Chitwan district	2017	Chicken meat	38	4.8 %	[84]
Kanchanpur district	2017	Fresh chicken meat	45	40 %	[74]
Nepal (various regions)	2019	Chicken meat (skin, flesh, and liver)	180 (60 each)	33.3 % overall and 26.7 % of isolates resistant to colistin (*mcr-*1 gene detected)	[85]

Several studies from the Chitwan district also highlight *E. coli* contamination in meat. In Bharatpur, 56 % of 100 chicken samples from wholesale and retail shops were contaminated, with a higher rate in fresh samples (72.8 %) [68]. Ranabhat et al. [83] reported a 58.1 % prevalence in 105 broiler meat samples, while Regmi et al. [84] found 33 % contamination in meat samples from chopping boards, though non-ESBL-producing strains. A study by Shrestha et al. [71] detected *E. coli* in 4.8 % of 38 chicken meat samples. Similarly, in a single study from western Nepal, 45 fresh chicken meat samples from Kanchanpur were analyzed, where 40 % (18/45) were contaminated with *E. coli* [74]. In addition to the high prevalence of *E. coli* across various regions of Nepal, the detection of antibiotic-resistant genes is particularly alarming. A study by Joshi et al. [85] examined 180 chicken meat samples (60 each of skin, flesh, and liver), isolating *E. coli* in 33.3 % (60/180) of the samples. Notably, 26.66 % (16/60) of these isolates were resistant to colistin and carried the *mcr*-1 gene, highlighting a serious public health concern.

#### 3.2.3 Campylobacter

*Campylobacter* species are major causes of human gastrointestinal infections globally, with *Campylobacter jejuni* and *Campylobacter coli* frequently causing gastroenteritis [86]. These bacteria are common in infants with diarrhea in developing countries like Nepal due to contaminated food or water [87]. Poultry, along with other domestic animals, are a major source and reservoir of these zoonotic pathogens, facilitating the transmission of campylobacteriosis to humans [88]. In Nepal, *Campylobacter* is one of the leading causes of foodborne infections, and antibiotic-resistant strains of this bacterium have been reported in poultry and pig carcasses [87,89,90]. Research on campylobacteriosis in Nepal is limited, largely because many cases go undiagnosed due to the lack of hospitalization requirements and the need for advanced laboratory procedures, which are not widely accessible. Ghimire et al. [90] analyzed 139 pork samples from the neck, ham, shoulder, and skin, finding a *Campylobacter* prevalence of 38.85 % (54/139). Of the isolates, 77.8 % were *C. coli* and 22.2 % were *C. jejuni*. Similarly, Bhattarai et al. [91] collected 400 retail broiler meat samples from Chitwan, with 44.8 % testing positive for *Campylobacter*. Among these, *C. jejuni* was the most prevalent at 80.4 %, followed by *C. coli* at 14.5 % and *C. lari* at 4.9 %. Additionally, 7.3 % of the samples were infected with multiple *Campylobacter* species. Moreover, Bhattarai et al. [89] sampled water from 200 slaughterhouse sites in Kathmandu and Rupandehi districts, finding a *Campylobacter* prevalence of 12 % in Rupandehi and 0 % in Kathmandu.

#### 3.2.4 Shigella

*Shigella* is a major cause of diarrhea, particularly dysentery, often referred to as bacillary dysentery. Among the four species of *Shigella*, serotype A (*Shigella dysenteriae*) and serotype B (*Shigella flexneri*) are the most commonly associated with shigellosis in Nepal [92]. Globally, changes in prevalent serogroups have been observed, and Nepal is no exception. While *S. dysenteriae* was the predominant species in Nepal during 2003 and 2004, *S. flexneri* has been the most prevalent since 2005 [[92], [93], [94]]. This global trend is mirrored in local studies. A study conducted in 45 samples of fresh chicken meat from Kanchanpur found that 17 samples (38 %) were contaminated with *Shigella* [74]. Similarly, in Dharan, 6 % of 50 meat samples (15 chicken, 15 pork, 10 buffalo, and 10 chevon) collected from local markets were tested positive for *Shigella*, with specific prevalence rates of 4 % in chicken, 0 % in pork, 2 % in buffalo, and 0 % in chevon [12]. Bantawa et al. [72] reported a 6 % positive rate for *Shigella* in 83 meat samples (33 chicken, 27 pork, 13 buffalo, and 10 chevon) from various meat shops in Dharan. Additionally, a study in the Kathmandu Valley revealed a 2.5 % prevalence of *Shigella* in 40 raw chicken meat samples from local markets [80].

#### 3.2.5 S. aureus

*S*. *aureus* is a significant foodborne pathogen found in both fresh and ready-to-eat foods, and it is associated with a range of infections globally [95]. Due to its strong adaptability, *S. aureus* can adjust to diverse environmental conditions and quickly develop resistance to nearly all antibiotics [96]. Recently, MRSA has gained significant attention due to its AMR to multiple antibiotics. In 2017, the WHO identified it as one of the 12 bacterial families representing the greatest threat to human health [97]. In a study, 57 % of 45 fresh chicken meat samples from Kanchanpur were tested positive for *S. aureus* [74]. Bantawa et al. [12] found that 68 % of the 50 meat samples from Dharan were positive, with prevalence rates of 53.33 % in chicken meat, 73.33 % in pork, 80 % in buffalo meat, and 70 % in chevon. A subsequent study reported a 68 % positive rate for *S. aureus* in 83 meat samples from Dharan [72]. In Bhaktapur, 8.6 % of 70 buffalo meat and 12.9 % of 70 chicken meat samples were positive for *S. aureus* [66]. In Biratnagar, 48.8 % of 80 meat samples (40 chicken and 40 mutton) were tested positive, with 52.5 % in chicken meat and 45 % in mutton [82]. Additionally, a study conducted by Devkota et al. [98], isolated 139 *S. aureus* strains (67.8 %) from 205 samples, including 71.2 % from eggs and 62.5 % from chicken meat, with an overall MRSA prevalence of 12.94 %. These findings underscore the urgent need for enhanced monitoring and control measures.

#### 3.2.6 Listeria monocytogenes

*L*. *monocytogenes*, the primary cause of listeriosis, is a significant emerging foodborne bacterial zoonotic pathogen of global importance. This bacterium has been found in meat, poultry, milk, cheese, other dairy products, and vegetables [99]. *L. monocytogenes* is a major food safety concern as it can cause disease in humans and can be transmitted through animal-derived food products [100]. Refrigeration doesn't inactivate or kill bacteria, so adequate cooking is of great importance [101]. Due to its high mortality rate, listeriosis and outbreaks caused by *L. monocytogenes* have a significant economic impact on public health and the food industry. This disease particularly affects young children (neonates) and the immunocompromised elderly population [102]. There are no published data on the direct presence of *Listeria* in milk and meat in Nepal. A study conducted at the College of Medical Sciences, Bharatpur, screened 234 antenatal mothers aged 17–39 years, who were between 7 and 36 weeks of gestation and exhibited flu-like symptoms, for *L. monocytogenes*. The study found a 16.7 % prevalence of *L. monocytogenes* (39/234), with the highest infection rate (53.1 %) observed among women aged 25 to 32, predominantly from urban areas. The study identified that the highest rates of listeriosis were associated with the consumption of meat (97.4 %, 38/39), fish (100 %, 39/39), non-pasteurized boiled milk (100 %, 39/39), and vegetables such as salad (82.1 %, 32/39) [103]. These findings highlight an urgent need for surveillance and control measures for *L. monocytogenes* in Nepal, particularly in high-risk food items and vulnerable populations.

### 3.3 Raw milk and meat consumption in Nepal

Nepal is a multi-cultural nation with diverse ethnic and cultural practices, many of which lack scientific validation. One such practice is the consumption of raw milk and meat. Every day, a significant quantity of inadequately pasteurized milk and dairy products is consumed in Nepal, and several reports have documented contamination of these products with harmful microorganisms [60]. A recent survey revealed that many individuals regularly consume raw milk [[60], [104]], increasing the risk of acquiring zoonotic foodborne illnesses. Additionally, in various regions, raw milk serves as the base for traditional products such as *dahi* (yogurt), *gheu* (dried butter), and *mohi* (buttermilk) [105], further elevating the risk of zoonotic disease transmission. While these dairy products are consumed across Nepal, they are particularly common in rural areas and among subsistence farmers [105].

Knowledge of zoonotic risks among livestock farmers in Nepal remains alarmingly low. A recent study of 380 livestock farmers found that only 1.6 % were aware of zoonotic brucellosis, and just 3.1 % knew about bovine TB [106]. These findings highlight a significant gap in awareness regarding zoonotic diseases. More than 80 % of Nepal's population adheres to Hinduism, with approximately 16 % identifying as *Chhetri* and 12 % as *Brahmin* [107]. Within Hindu religious practices, especially among *Chhetri* and *Brahmin* communities, consuming *Panchamrit*—a sacred mixture of five ingredients, including raw cow milk—is customary during almost all religious ceremonies (*puja*) [108]. Although typically only 1–2 spoonfuls of *Panchamrit* are consumed on these occasions, the risk of zoonotic transmission cannot be overlooked, particularly since around 28 % of Nepalese people consume *Panchamrit* at least once annually.

Although properly cooked meat is commonly consumed in Nepal, a significant portion of the population also consumes raw or undercooked meat products, contributing to zoonotic disease risks. Various ethnic communities maintain unique traditions involving raw meat consumption. For example, the Newar community prepares indigenous dishes such as *Kachela* (minced raw buffalo meat) and *Chhoyla* (smoked buffalo meat). Similarly, *Sekuwa*—roasted pork, chicken, or chevon cooked over a natural wood fire—is often consumed semi-cooked. The *Dum* communities in southeastern Nepal traditionally consume raw or undercooked meat and viscera from scavenging pigs during religious and social festivals. In the hilly and Himalayan regions of far-western Nepal, people consume *Kachmali*, which includes raw chevon with body parts such as ears, tail, liver, kidney, tongue, skin, brain, sternum, and testicles. Additionally, the Indo-Aryan ethnic group in the far-western region frequently consumes raw wild boar meat, known as *Bade.* Cultural practices such as drinking fresh raw yak blood, believed to have medicinal benefits, are also observed among the *Sherpa* community in Solukhumbu and the Thakali people in Mustang [109].

Although several foodborne diseases in livestock capable of transmitting to humans through the consumption of raw or undercooked meat have been documented in Nepal, limited research has assessed the actual burden of these infections within the population.

Few studies are conducted for helminths and protozoa, and among the few studies available, serological research conducted in the western regions (Dang and Accham) reported a high prevalence of *Toxoplasma gondii* infection among the Indo-Aryan group, particularly those with raw meat-eating habits [110]. Furthermore, the frequent diagnosis of neurocysticercosis, primarily linked to consuming undercooked pork, underscores the urgent need for additional research and public health interventions [109].

Further studies are essential to identify the risk factors and prevalence of foodborne bacterial pathogens across various types of milk and meat in Nepal, especially among communities and ethnic groups that consume raw milk, meat, and blood.

### 3.4 Lack of regulatory framework and enforcement

In Nepal, the Department of Food Technology and Quality Control has set legal standards for milk and dairy products, including microbiological, chemical, and physical criteria to ensure their safety for consumption. The National Dairy Development Board introduced the *Code of Practice for the Dairy Industry 2061 (2004)*, outlining six criteria for standardizing milk and dairy products: organoleptic, clot on boiling (COB), alcohol, milk fat (FAT) and solids not fat (SNF), adulteration, phosphate, and microbial and coliform testing [111]. Similarly, the *Food Act (1967)* and the *Animal Slaughterhouse and Meat Inspection Act (1999)* are the primary legal frameworks for meat safety in Nepal [14]. However, these laws fail to address modern food safety challenges due to factors such as lack of risk assessment principles, insufficient resources and infrastructure, inadequate facilities for food contamination analysis, and limited stakeholder awareness [112]. The *Food Safety Policy 2076* was approved by the government on 23 June 2019, marking an initial step toward improving food safety [113]. In May 2021, Kathmandu Metropolitan City established eight slaughterhouses and cold storage facilities to handle large quantities of meat. In 2019, Heifer International Nepal partnered with five local governments—Pokhara, Biratnagar, Bharatpur, Butwal, and Kolhapur to develop modern abattoirs [114]. Dairy development is overseen by the Ministry of Agriculture and Livestock Development (MoALD) and implemented through the National Dairy Development Board (NDDB), the country's leading autonomous body for dairy development.

**Fig. 3:**
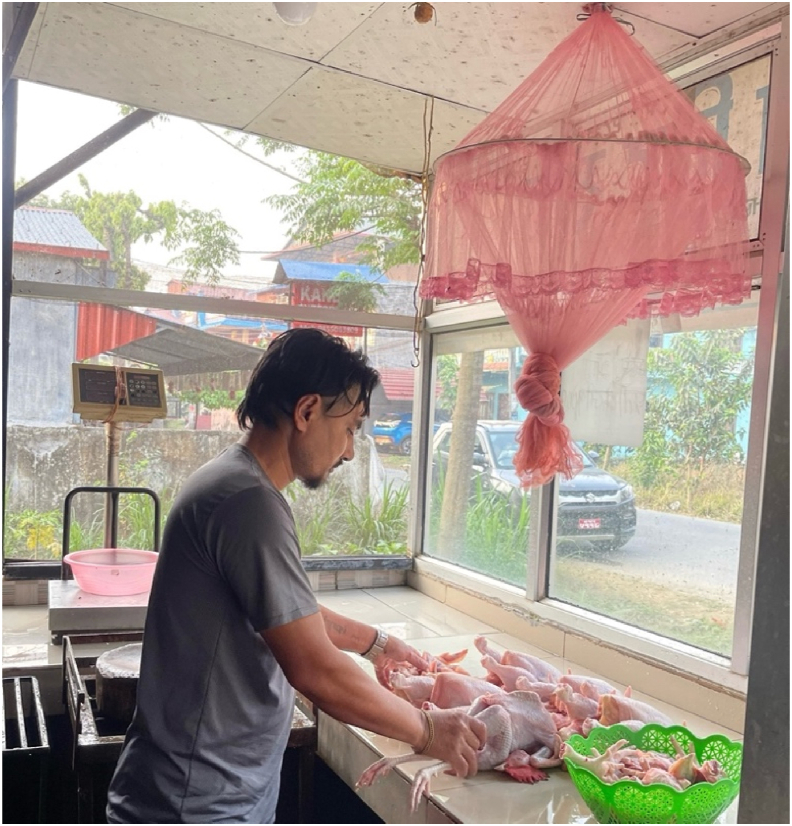
A local poultry meat shop (butcher shop) in Bharatpur, Chitwan, Nepal, preparing chicken for sale. While this setup reflects the accessibility and community-based nature of meat distribution in the country, it also highlights opportunities for improvement in hygiene practices and infrastructure. Enhancing cold storage, introducing protective equipment, and providing training on sanitary meat handling can significantly uplift food safety standards and support the livelihoods of small-scale meat retailers.

In Nepal, meat for consumption is sourced differently in urban and rural areas. In urban centers, meat is typically purchased from local butchers (Fig. 3), while in villages, communal animal slaughtering is common, and the meat is shared among several families on a cost-sharing basis. Lack of enforcement of meat inspection regulations and the absence of proper slaughterhouse facilities lead to unsanitary practices. Animals are slaughtered in unregulated spaces, including the premises of meat shops, streets, riversides, backyards, and open pasturelands, often lacking sanitation facilities and formal antemortem or postmortem examinations, increasing the potential risk of zoonotic and foodborne disease transmission. Additionally, inadequate sanitary and hygiene practices in butcheries during bleeding, handling, processing, retailing, and storage contribute to the microbial contamination of raw meat.

The formal or organized sector accounts for only about 20 % of the total annual milk production in Nepal, while the remaining 80 % is handled by the informal sector, where milk is collected, processed, and distributed through traditional channels with minimal regulation and oversight [115]. This widespread reliance on informal markets presents significant challenges for ensuring milk safety, quality control, and public health protection.

Milk handling and distribution in Nepal present numerous challenges that significantly threaten public health. Collection, transportation, and sale of raw milk frequently occur without proper refrigeration or adherence to hygiene standards, leading to a high risk of microbial contamination. In rural areas, farmers often collect milk from multiple households and pool it together, increasing the likelihood of contamination. If even a small portion of the pooled milk contains pathogens, the entire batch becomes unsafe for consumption. This risk is further amplified by the lack of adequate screening and testing facilities to ensure milk safety before pooling and distribution [115].

Transporting raw milk over long distances without refrigeration remains a critical issue. In Nepal, milk is commonly transported in non-refrigerated vehicles, exposing it to fluctuating temperatures and environmental conditions. Warm climate in the Terai region accelerates bacterial growth in unrefrigerated milk, making it unsafe by the time it reaches consumers. Furthermore, transporting milk in open containers, often on the backs of motorbikes or bicycles, exposes it to dust, dirt, and other contaminants, compounding the risk of microbial contamination.

One of the most significant shortcomings in Nepal's milk distribution system is the limited use of pasteurization, a crucial process that eliminates harmful bacteria and pathogens. In many rural areas, farmers sell milk directly to consumers without pasteurization, substantially increasing the risk of zoonotic diseases such as brucellosis and tuberculosis [16].

Infrastructural deficiencies further hinder milk safety. The absence of cold storage facilities at collection points and during transportation means that milk is often stored at ambient temperatures, which are unsuitable for preserving its safety and quality. Addressing these gaps requires investment in cold chain infrastructure, including refrigerated collection centers and transport vehicles, to reduce contamination risks [116].

Moreover, Nepal's regulatory framework for milk safety requires strengthening. There remains a significant disconnection between policies as written and their implementation in practice [117]. Implementing clear guidelines and standards for milk collection, transportation, and processing, alongside rigorous enforcement through regular inspections and quality control, is essential to safeguarding public health. However, small-scale farmers and local vendors often lack the financial resources to invest in proper storage and transportation equipment, forcing them to rely on traditional practices that fall short of modern hygiene standards.

### 3.5 Antibiotic residues in milk and meat

Antibiotics, whether naturally derived or synthetically produced, play a vital role in treating and preventing diseases in both humans and animals. However, their misuse, particularly as growth promoters in food animals and their indiscriminate administration without veterinary oversight, has led to two major concerns: the persistence of antibiotic residues in food products and the rise of AMR, both of which pose significant risks to food safety and human health [118].

In Nepal, several studies have reported the presence of antibiotic residues in milk and meat. Commonly used antibiotics in the livestock sector include cephalosporins, aminoglycosides, fluoroquinolones, macrolides, penicillin, sulfonamides, nitrofurans, tiamulin, and tetracyclines [118]. In 2019, the animal industry in Nepal consumed approximately 48 t of antibiotics. This included 9.1 t of third- and fourth-generation cephalosporins, 9.7 t of tetracyclines, 6.5 t of fluoroquinolones, and various quantities of other antibiotic classes [119].

The presence of antibiotic residues in milk has been well documented. Fresh milk samples collected from different areas of the Kathmandu Valley and tested by the Veterinary Standards and Drug Regulatory Laboratory reported that 5 % were positive for antibiotic residues, with sulfonamides being the most common [120]. Similarly, milk samples from five provinces of Nepal, including Bagmati, Gandaki, Koshi, Lumbini, and Madesh, tested in 2021 and 2022, showed a prevalence of 1.6 % for antibiotic residues [121]. Higher levels were reported in districts such as Kailali, Kaski, and Nuwakot, where residues of tetracyclines, streptomycin, and sulfonamides ranged from 44 % to 75 %, with sulfonamides frequently exceeding the national maximum residue limits [122]. Additionally, milk from individual farmers, cottage dairies, and organized dairies in the Kathmandu Valley contained sulfonamide and penicillin residues above the national limits [123].

Meat products have also shown significant contamination. In Kavrepalanchok and Kailali districts, over 20 % of tested meat samples from poultry, goats, buffaloes, and pigs contained residues of penicillin, tetracycline, aminoglycosides, macrolides, and sulfonamides [124]. Broiler meat samples from Kathmandu Metropolitan City showed an even higher prevalence of antibiotic residues at 28.25 % [125]. Quinolone residues, which belong to a critically important class of antibiotics, were detected in poultry meat and eggs from the Kathmandu Valley [126]. Furthermore, chicken meat from Kailali, Kaski, and Nuwakot districts contained residues of tetracyclines, sulfonamides, penicillin, gentamycin, and streptomycin, with contamination rates ranging from 50 % to 83 % [122].

These findings emphasize the widespread presence of antibiotic residues in milk and meat across Nepal. This contamination presents a serious threat to public health and food safety, highlighting the urgent need for stronger regulatory enforcement and responsible use of antibiotics in livestock production systems.

### 3.6 Limited public awareness and education

Ensuring the safety of animal-sourced foods such as meat and milk presents an important opportunity for improving public health in Nepal. However, challenges persist due to limited awareness and education among producers, traders, and consumers. For instance, dairy farmers are often unaware of proper barn hygiene, sanitation of milking equipment, and the importance of producing milk under hygienic conditions [127]. In many cases, milk becomes contaminated at the farm level, largely due to exposure to manure and poor hygiene practices within barns and among farmers. Essential practices such as handwashing, cleaning of teats and udders before milking, and proper sanitization of milking vessels and transport containers are frequently neglected due to a lack of training. As a result, milk can become contaminated with vegetation, soil, bedding materials, and microorganisms originating from these sources [128]. Transportation of milk also poses significant risks. Milk is commonly carried to collection centers in plastic containers, and many farmers are unaware that improper cleaning and sanitization of these containers can harbor harmful pathogens, increasing the risk of milk-borne diseases [129].

Similarly, meat safety is compromised by inadequate education and training among butchers and meat handlers regarding proper sanitation practices. Many meat sellers lack knowledge about the importance of disinfecting hands, tools, clothing, and using protective gear during meat handling and selling [130]. Furthermore, common practices such as displaying meat without refrigeration, maintaining poor cleanliness of premises, and using unclean water contribute to widespread contamination. These conditions attract flies, insects, and dirt, exacerbating the risk of meat contamination. A study conducted in retail meat shops within Butwal Municipality found that 70.0 % of meat handlers had no formal education, while 82.5 % had never received any training in meat handling or hygiene practices [131].

In addition to these food safety concerns, there is limited awareness of livestock-associated zoonoses among smallholder farmers in Nepal [132]. Women, who comprise most of the agricultural workforce and manage most livestock husbandry tasks, are often excluded from formal livestock education and training programs. This lack of access to education limits their understanding of zoonotic diseases originating from livestock [[133], [134]].

### 3.7 Inadequate infrastructure

Nepal holds significant potential to enhance food safety by improving infrastructure for hygienic meat and milk production. However, the country continues to face major challenges in this regard. There is a shortage of modern slaughterhouses, and many existing facilities are either non-operational or lack the basic infrastructure needed to ensure hygienic meat production [112]. In most cases, slaughtering takes place in environments with poor hygiene and safety standards, resulting in a high risk of contamination [135].

To address these concerns and safeguard consumer health, the Government of Nepal introduced the *Slaughterhouse and Meat Inspection Act 1999*. Despite this policy framework, the act has yet to be fully implemented [131]. Key provisions, such as ante- and post-mortem examinations of animals and regular inspections of meat facilities by government-appointed inspectors, are rarely enforced [136]. Several factors hinder effective implementation, including inadequate infrastructure, a shortage of trained personnel, weak regulatory enforcement, and limited financial resources. Butcheries remain the primary outlets for meat sales in Nepal, yet many lack even the most basic facilities for hygienic meat handling [131]. Essential infrastructure, such as tiled or marble surfaces for walls and floors, proper ventilation, air conditioning, reliable refrigeration, stainless steel equipment, effective waste disposal systems, and an uninterrupted power supply, is often missing. These deficiencies highlight an urgent need to upgrade infrastructure and enforce food safety standards to promote hygienic meat production.

**Fig. 4:**
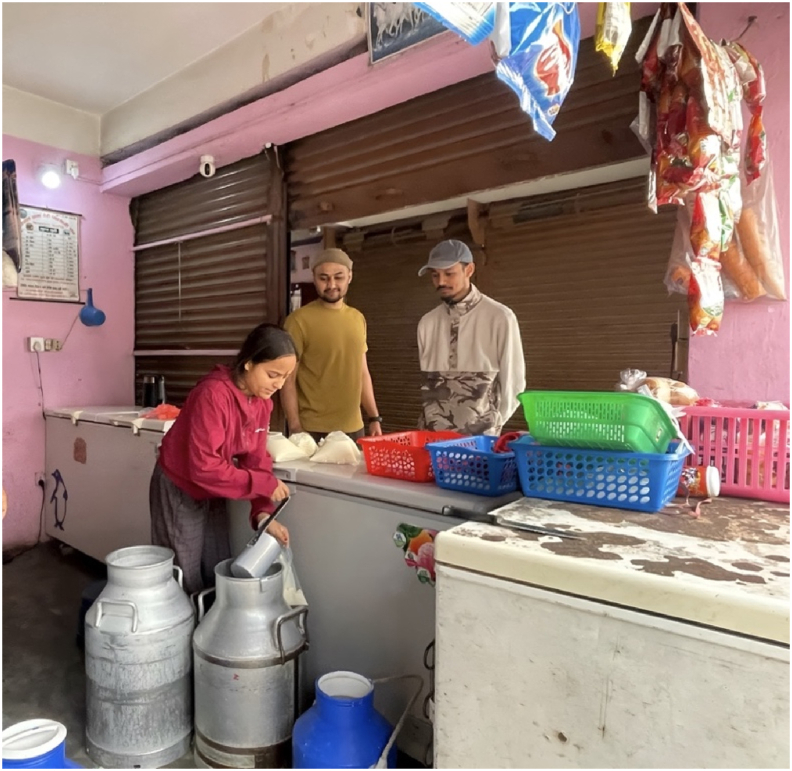
A local dairy (together with a grocery) shop in Bharatpur, Chitwan, Nepal, where raw milk is being poured into a plastic bag from the large metal container for direct sale to consumers. This setup demonstrates the community-based milk supply system that plays a vital role in meeting local dairy needs. While traditional methods remain prevalent, the scene also underscores the opportunity to strengthen milk hygiene and safety through the adoption of improved handling practices, cold chain infrastructure, and training for milk handlers.

The dairy sector faces similar constraints. Milk production in Nepal is primarily carried out by small-scale, scattered, and unorganized farmers, who often operate without the necessary infrastructure for proper collection, transportation, processing, and marketing [115]. The absence of specialized aseptic utensils for milk collection and transportation, combined with a lack of cold chain infrastructure from farms to collection centers and local retail outlets (Fig. 4), facilitates the growth of spoilage organisms and deteriorates milk quality. Furthermore, unpaved and rough roads in rural areas cause turbulence and delays during milk transportation, contributing to fat breakdown and the development of off-flavors [127].

Post-collection storage is another challenge. Many areas lack sufficient cold storage facilities, and limited access to processing plants further exacerbates the issue. Additionally, restricted access to veterinary services in rural areas, where most milk is produced, affects both milk quality and production. Together, these factors compromise the safety and hygiene of milk in Nepal's supply chain, underscoring the need for infrastructure development and improved veterinary support to ensure the production of safe, and high-quality milk [115].

## 4 Strategies to prevent milk-borne and meat-borne illnesses in Nepal

### 4.1 Upholding stringent hygiene practices in the meat and milk supply chain

It is essential to maintain rigorous hygiene protocols at all stages of the meat supply chain, including slaughtering, processing, storage, distribution, and retail [131]. Measures should include maintaining regular cleaning and disinfection of tools and facilities, proper disposal of animal waste, and the use of cold storage to prevent microbial growth. Enforcing strict personal hygiene practices for workers, such as mandatory handwashing after handling dirty objects like money, using the toilet, or touching the face, along with requiring them to wear clean work clothes and protective gear such as hair coverings, beard nets, and gloves, can significantly reduce the risk of contamination. Ensuring safe handling and protective measures during slaughtering, processing, transportation and sales will minimize contamination risks.

**Fig. 5:**
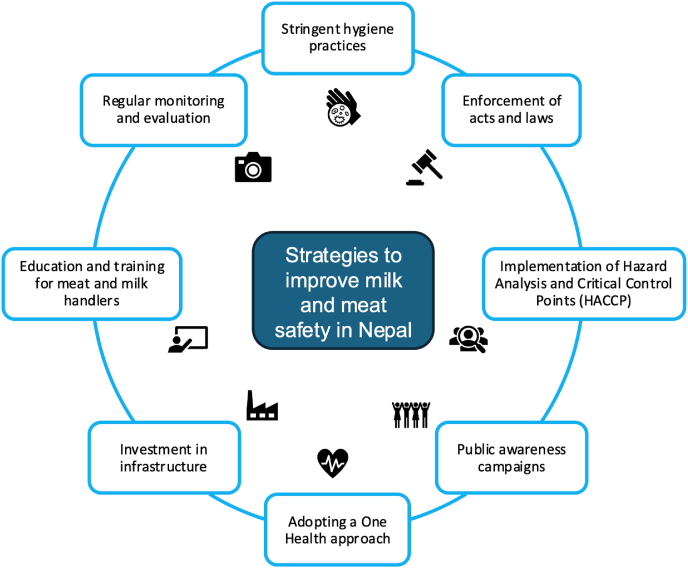
Strategies to improve milk and meat safety in Nepal.

Similarly, strict food safety standards and regulations should be enforced for quality milk production and distribution in Nepal (Fig. 5) [116]. This includes the adoption of clean milking practices by farmers, such as washing hands, maintaining udder hygiene, and using clean milking equipment to prevent microbial contamination [137]. Provision of refrigeration facilities, especially in rural areas, and the establishment of a cold chain system, such as insulated containers during transportation, should be implemented. Containers for milk storage and transportation should be clean, regularly sanitized, and the use of food-grade containers should be made mandatory [138]. Facilities for regular microbial and quality testing at collection centers and dairy processing plants should be established.

### 4.2 Enforcement of the *Animal Slaughter and Meat Inspection Act*

The *Animal Slaughter and Meat Inspection Act* in Nepal serves as a crucial regulatory framework to ensure the safety and hygiene of meat products. The act mandates compulsory ante-mortem and post-mortem inspection of animals, proper slaughtering facilities, and hygienic handling of meat products [112]. Although the act was passed in 1999 and regulated in 2001, it has yet to be successfully enforced. Strict implementation of this act is crucial to ensuring the production of safe, high-quality meat for public consumption. The government and private sector should invest in establishing certified slaughterhouses equipped with modern facilities and ensure regular inspections of these meat enterprises by government-appointed meat inspectors to enforce compliance with regulations [136]. Provision of strict penalties should be enforced for non-compliance and unsafe practices, along with the introduction of traceability systems to enhance accountability throughout the supply chain [139]. Collaboration between government agencies, local authorities, and private stakeholders can further enhance the implementation of the act, ensuring safer meat production and distribution across Nepal.

### 4.3 Education and training for meat and milk handlers

Proper education and training for meat and milk handlers are essential to ensure food safety, hygiene, and quality throughout the supply chain. Lack of awareness and improper handling practices can lead to microbial contamination of meat and milk, posing serious health risks to consumers [140].

Butchers and meat handlers in Nepal should receive training on best practices for hygiene and food safety. These programs should cover topics such as safe handling, proper storage, thorough cleaning of tools and surfaces, and the use of personal protective equipment (PPE) [141]. Similarly, milk handlers should be trained in clean milking techniques, proper pasteurization, and safe storage to prevent microbial contamination [127]. Government agencies, along with food safety authorities should implement mandatory training and certification programs. Certification programs can validate their skills and incentivize adherence to hygiene standards [142]. Regular workshops and awareness campaigns can further reinforce safe handling and sanitation practices among food industry personnel. Ensuring all individuals involved in meat and milk processing receive proper training and education will contribute to improved public health and food safety standards in Nepal. A significant association was observed between maintaining sanitation and hygiene practices among meat sellers in Nepal compared to those who had not received any kind of training [[141], [143]]. Training food handlers on the importance of personal hygiene and educating them on better food safety practices play an important role in reducing the risk of foodborne illnesses and ensuring safe food products for consumers [144].

### 4.4 Public awareness campaigns

Public awareness campaigns should be launched to educate communities about the risks of consuming raw or undercooked meat and milk in Nepal. These campaigns should emphasize safe consumption practices, such as thorough cooking of meat and pasteurization of milk. In a recent study [140] conducted in various districts of Nepal among 280 livestock farmers, 11 % of farmers reported consuming undercooked or raw meat, while 19 % reported consuming raw or boiled milk. Most farmers and consumers in Nepal are unaware of the potential risks of zoonotic diseases transmitted through the consumption of raw or contaminated meat, and the practice of consuming sick or recently dead animals further exacerbates the situation [145]. A study conducted among 380 respondents in the Manang, Tanahun, and Nawalpur districts of Gandaki Province reported 17 % of participants were practicing the consumption of sick or dead animals, highlighting the need for increased awareness and food safety measures [106]. Messages tailored for both urban and rural populations can help address cultural traditions and practices while promoting safer food consumption and hygiene habits to reduce health risks [146]. For example, an educational workshop conducted in the United States effectively reduced the incidence of *Salmonella* Typhimurium, which was associated with consuming fresh cheese [147]. Similarly, a study conducted in Tanzania found that narrative messages can effectively promote healthy hygiene practices and improve milk quality in a pastoral community, even when cultural norms conflict with best health practices [148].

**Fig. 6:**
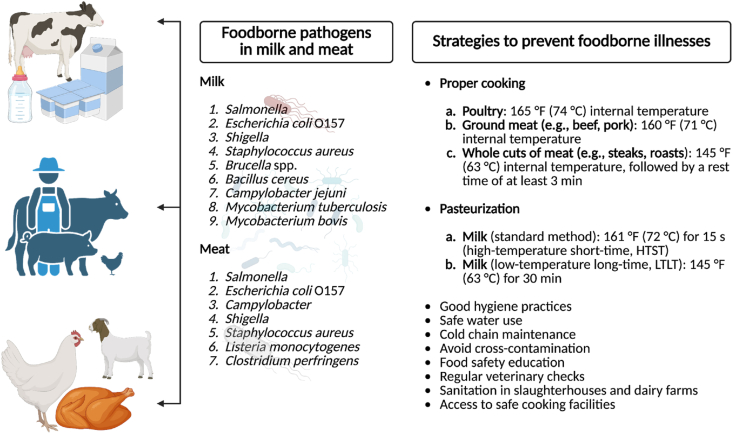
Foodborne pathogens in milk and meat and strategies to prevent foodborne illnesses from these pathogens.

In Nepal, similar approaches should be implemented to enhance public awareness and encourage behavior change. Schools need to incorporate food safety education into their curriculum. Similarly, local leaders can play a key role in advocating for safe consumption practices within their communities, and media platforms can be utilized to disseminate informative campaigns on the risks of consuming raw or contaminated meat and milk. Besides these, information can be shared through mass media such as social media, radio, and television, along with printed materials like posters and brochures [111]. By leveraging these channels, awareness initiatives can effectively reach a wider audience, fostering long-term improvements in food safety and public health. Educational initiatives should be customized to meet the specific needs of urban and rural populations. For rural areas, focus on safe livestock handling, traditional slaughter practices, and proper meat storage. For urban populations, emphasize the importance of purchasing meat from certified vendors and safe preparation methods at home. These programs should respect cultural practices while promoting food safety. Consumers should be informed about the importance of boiling or pasteurizing milk, maintaining proper refrigeration for milk and meat, practicing hygienic handling of raw foods, thoroughly cooking meat, avoiding unpasteurized dairy products, choosing trusted sources, and adopting safe food practices to reduce the risk of foodborne illnesses (Fig. 6).

### 4.5 Regular monitoring and evaluation

Regular monitoring and evaluation by health authorities and local governments are crucial to preventing meat-borne and milk-borne illnesses in Nepal. Regular inspections of slaughterhouses, meat and dairy shops, milk parlors, and the entire meat and milk supply chain are essential to ensure compliance with hygiene standards. For hygienic meat production, authorities should proactively carry out regular monitoring of abattoirs and butcheries to enforce sanitary practices, including cleanliness of facilities, proper handling and storage of meat, waste disposal practices, and adherence to personal hygiene protocols among workers [149]. Similarly, for milk production, regular inspections should focus on ensuring that dairy farms, milk parlors, and milk collection centers maintain proper milking hygiene, safe storage temperatures, and sanitation of milking equipment to prevent contamination [150]. A license should be mandatory for all meat and dairy producers, sellers, and processors, with strict penalties enforced for those selling products without a valid license or violating food safety regulations [112]. Samples of meat and milk products should be collected for laboratory testing to detect adulterants and contaminants, and it should be prohibited to produce, sell, distribute, export, or import any adulterated or sub-standard food, or hold such food for any of these purposes [151]. Furthermore, proper labelling should be mandatory for all milk and meat products, including clear information on the name of the food, ingredients, the manufacturer's name and address, net quantity, production and expiration dates, nutritional content, and storage instructions to ensure consumer safety and transparency [152]. Authorities should establish a feedback system for consumers to report food safety concerns, allowing prompt action against violations. They should also publicly share periodic reports on inspection results and corrective measures to ensure transparency and enhance public awareness of food safety.

### 4.6 Investment in infrastructure for meat and milk processing and storage

Developing robust infrastructure for meat and milk processing and storage is essential to ensuring food safety, quality, and sustainability in Nepal's livestock sector. Large-scale meat and dairy industries are lacking in Nepal, with the sector primarily consisting of small-scale suppliers, local butcheries, and dairy farmers. In Nepal, only eight slaughterhouses have been registered to date; however, almost all of them are either non-operational or in poor condition [153]. Similarly, the dairy sector faces challenges such as inadequate milk collection centers, limited cold chain facilities, and poor processing infrastructure, leading to significant post-harvest losses and compromised milk quality [127].

The negligence from the government authorities, including a lack of funding, inadequate policies, and poor enforcement of regulations, has further hindered the development of a structured and hygienic meat [112,135] and dairy industry [116]. Investments should focus on upgrading and ensuring the functionality of already established slaughterhouses and dairy processing facilities, as well as constructing additional ones in different districts of Nepal based on need and demand. All three tiers of government in Nepal should allocate sufficient budget and also engage relevant stakeholders to invest in the development, maintenance, and modernization of the meat and milk processing infrastructure. For sustainability, these facilities should be established away from densely populated areas and natural environmental resources, in both rural and urban centers, to minimize the risk of contamination and pollution in residential environments and prevent contamination of water supplies [149]. The slaughterhouse facilities must be equipped with proper sewage and waste disposal systems, a clean water supply, cold storage, effective vector control measures, and must maintain strict hygiene protocols to ensure food safety and prevent contamination of meat, the environment, and the related food products [154]. Similarly, investments should be made in expanding cold chain infrastructure, including refrigerated transportation and storage facilities, to maintain milk quality, prevent spoilage, and reduce losses in the dairy sector [150]. The government should implement a public-private partnership model to invest in milk and meat infrastructure. Additionally, it should provide grants and subsidies to private organizations and cooperatives to support their establishment and ensure long-term, sustainable operations.

Besides this, the government should invest in improving road networks connecting slaughterhouses and milk collection/processing centers to facilitate the transportation of live animals and the distribution of processed meat [140]. Furthermore, resources should be allocated to establishing and upgrading laboratories with modern diagnostic equipment and skilled personnel for routine meat and milk testing.

### 4.7 Implementation of Hazard Analysis and Critical Control Points (HACCP) protocol

The implementation of HACCP protocols is crucial for systematically identifying and mitigating risks at critical points, ensuring the production of safe meat, milk, and their products. This approach integrates good hygienic practices and standard sanitation operating procedures across all stages of production, processing, distribution, and storage in the meat [155] and dairy [156] supply chain.

In the meat industry, contamination primarily occurs during processing when meat comes into contact with equipment, food handlers, and environmental factors [157]. The risk of contamination is particularly high through meat handlers’ hands and clothing, utensils (such as knives, saws, and mincers), cutting boards, and the transfer of bacteria from the meat surface to its internal parts during cutting and deboning [158,159]. To mitigate these risks, implementing a HACCP plan requires adhering to key prerequisites, including sanitary facility design, water quality management, proper sanitation of food contact surfaces, strict personal hygiene for food handlers, regular disinfection of utensils and equipment, effective pest control, proper waste disposal, and ensuring animal cleanliness [160]. Additionally, monitoring temperature controls during storage and distribution is crucial to preventing microbial growth and protecting meat from spoilage organisms throughout the supply chain [161].

Similar to the meat industry, the dairy sector requires HACCP implementation at various stages, including milk production on farms, processing in dairy facilities, and the storage and transportation of milk and its products to ensure safety, quality, and contamination control. The application of HACCP on dairy farms ensures safe and hygienic milk production, while also improving animal welfare and protecting the environment [162]. This can be done by monitoring major contamination routes, including infected animals, the hygiene and sanitation of farm workers, cleanliness of milking equipment, and water supplies for cleaning, and overall farm sanitation practices [163]. During dairy processing, HACCP should be implemented by identifying critical control points and control points at key stages, including milk reception, pasteurization, cooling, clean-in-place systems, drains inside the plant, dairy processing lines, packaging materials, and storage temperature, ensuring proper hygiene and product safety [164]. Furthermore, during transportation, HACCP should be implemented to ensure that raw milk is moved to processing plants in clean and disinfected tankers as quickly as possible. The tankers must be cleaned and disinfected immediately after unloading, and precautions should be taken to ensure that the driver is free from infectious diseases and follows strict hygiene practices throughout the process [156].

Besides this, comprehensive training on HACCP principles is essential for HACCP teams, managers, and food handlers, with the training being adjusted according to their technical expertise and the specific responsibilities they carry within the HACCP system [165]. Similarly, resources such as an adequate budget, manpower, monitoring equipment, and training facilities should be provided to the person in charge (supervisor) to ensure that an effective HACCP plan can be developed and implemented successfully. However, HACCP has not yet been made mandatory and is seldom applied by food producers, processors, and handlers in Nepal, and the government has yet to enforce regulations that would promote its widespread implementation by the industry [112].

### 4.8 Adopting a One Health approach

**Fig. 7:**
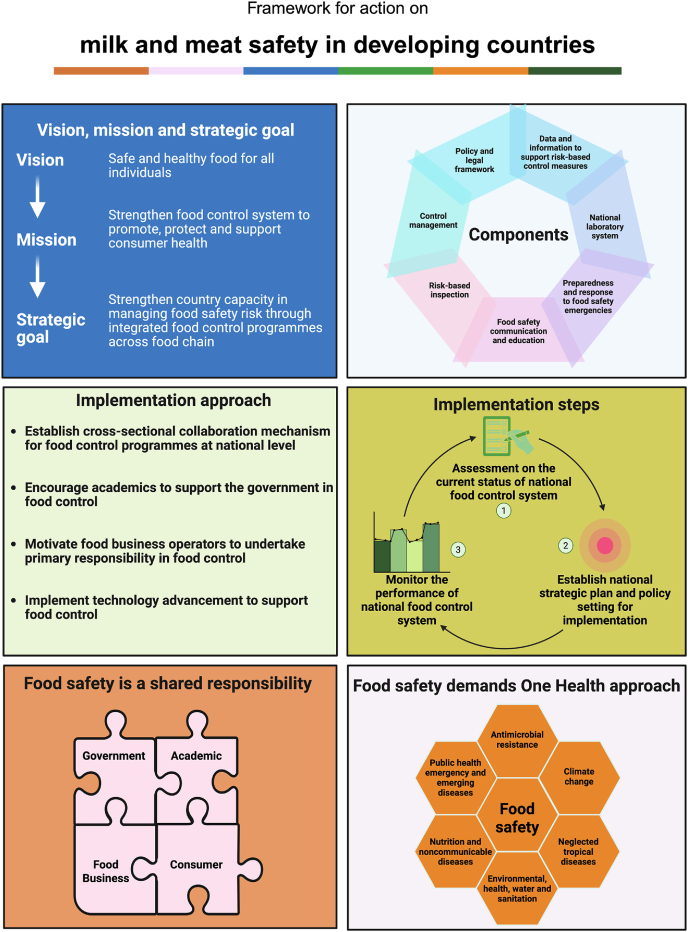
Model framework for action on milk and meat safety in developing countries like Nepal. This framework is modified from the framework provided by the World Health Organization for action on food safety in the Southeast Asia.

A One Health approach, integrating human, animal, and environmental health, is essential for managing meat- and milk-borne illnesses in Nepal. As human-animal interdependence intensifies within the global food system, associated health and environmental risks also increase [166]. Tackling foodborne diseases requires proactive, multi-disciplinary strategies under the One Health framework to develop effective and sustainable solutions (Fig. 7) [167]. Globally, approximately 12 % of diseases are linked to animal-source foods, with 4 % attributed to unsafe milk and milk products [168]. Key contributors to milk-borne illnesses include contamination during production, processing, transportation, and storage; zoonotic diseases in dairy herds; and consumption of unpasteurized milk [22].

To improve milk and meat safety, One Health strategies should include livestock vaccination, improved farm sanitation, hygienic dairy and meat handling practices, strict quality control, and widespread public health education [169]. Antibiotics overuse in the livestock and dairy sectors also fuels AMR, posing a growing global health threat. Regulatory frameworks must promote the prudent use of antibiotics to protect animal welfare, food safety, and human health [170]. Meat-borne diseases, often resulting from contaminated meat, remain a major concern worldwide. Pathogens can originate from infected animals, unhygienic environments, or improper handling and cross-contamination during processing [171]. Applying One Health across the meat and milk supply chains from farm to table can significantly reduce these risks [7].

To implement One Health approaches effectively in Nepal, specific actions must be taken. Authorities should establish joint surveillance of zoonotic pathogens, enforce environmental health measures such as safe water use and waste management, and ensure farm-level biosecurity and hygiene. Antibiotic stewardship programs should be strengthened, and consumers must be educated on safe food practices. A coordinated, multi-sectoral effort involving the Government of Nepal, veterinary and public health agencies, international organizations, donors, researchers, and communities is crucial to translating the One Health concept into concrete improvements in food safety [[134], [172]].

## 5 Conclusion

Milk and meat safety remain crucial public health concerns in Nepal due to persistent challenges such as poor hygiene practices, inadequate infrastructure, and weak regulatory enforcement. Despite the existence of legal frameworks, gaps in implementation continue to undermine food safety efforts. The predominance of informal sectors in milk and meat production further exacerbates contamination risks, posing serious health threats to consumers. Addressing these issues requires coordinated actions, including investment in modern slaughterhouses, temperature-controlled transport, robust surveillance systems, and capacity-building initiatives. Public awareness and education on safe food handling, coupled with stronger regulatory oversight, are essential steps toward ensuring safer food supplies. A One Health approach, integrating animal, human, and environmental health sectors, is crucial for sustainable improvements in food safety. Strengthening these areas will help mitigate the risks associated with milk and meat consumption in Nepal and contribute to better public health outcomes.

## CRediT authorship contribution statement

**Deepak Subedi:** Writing – review & editing, Writing – original draft, Supervision, Data curation, Visualization, Methodology, Conceptualization. **Sameer Thakur:** Writing – original draft, Data curation, Writing – review & editing, Visualization, Conceptualization. **Anil Gautam:** Writing – original draft, Data curation, Writing – review & editing, Investigation. **Madhav Poudel:** Writing – original draft, Data curation, Writing – review & editing, Investigation. **Sumit Jyoti:** Writing – original draft, Data curation, Writing – review & editing, Methodology. **Abhinandan Devkota:** Writing – original draft, Conceptualization, Writing – review & editing, Investigation. **Milan Kandel:** Writing – original draft, Data curation, Writing – review & editing, Investigation. **Ananda Tiwari:** Writing – review & editing, Methodology, Data curation, Writing – original draft, Investigation.

## Informed consent statement

Verbal informed consent was obtained from the individuals for the inclusion of their images in this publication.

## Ethics statement

The authors have nothing to report.

## Funding

The authors did not receive specific funding for this work.

## Declaration of competing interest

The authors declare no conflicts of interest.
